# PPAR-γ Agonist GW1929 Targeted to Macrophages with Dendrimer–Graphene Nanostars Reduces Liver Fibrosis and Inflammation

**DOI:** 10.3390/pharmaceutics15051452

**Published:** 2023-05-10

**Authors:** Alazne Moreno-Lanceta, Mireia Medrano-Bosch, Blanca Simón-Codina, Montserrat Barber-González, Wladimiro Jiménez, Pedro Melgar-Lesmes

**Affiliations:** 1 Department of Biomedicine, School of Medicine, University of Barcelona, 08036 Barcelona, Spain; amorenol@recerca.clinic.cat (A.M.-L.);; 2 Biochemistry and Molecular Genetics Service, Hospital Clínic Universitari, Instituto de Investigaciones Biomédicas August Pi i Sunyer (IDIBAPS), Centro de Investigación Biomédica en Red de Enfermedades Hepáticas y Digestivas (CIBERehd), 08036 Barcelona, Spain; 3 Institute for Medical Engineering and Science, Massachusetts Institute of Technology, Cambridge, MA 02139, USA

**Keywords:** liver, inflammation, fibrosis, graphene nanostars

## Abstract

Macrophages play essential roles during the progression of chronic liver disease. They actively participate in the response to liver damage and in the balance between fibrogenesis and regression. The activation of the PPARγ nuclear receptor in macrophages has traditionally been associated with an anti-inflammatory phenotype. However, there are no PPARγ agonists with high selectivity for macrophages, and the use of full agonists is generally discouraged due to severe side effects. We designed dendrimer–graphene nanostars linked to a low dose of the GW1929 PPARγ agonist (DGNS-GW) for the selective activation of PPARγ in macrophages in fibrotic livers. DGNS-GW preferentially accumulated in inflammatory macrophages in vitro and attenuated macrophage pro-inflammatory phenotype. The treatment with DGNS-GW in fibrotic mice efficiently activated liver PPARγ signaling and promoted a macrophage switch from pro-inflammatory M1 to anti-inflammatory M2 phenotype. The reduction of hepatic inflammation was associated with a significant reduction in hepatic fibrosis but did not alter liver function or hepatic stellate cell activation. The therapeutic antifibrotic utility of DGNS-GW was attributed to an increased expression of hepatic metalloproteinases that allowed extracellular matrix remodeling. In conclusion, the selective activation of PPARγ in hepatic macrophages with DGNS-GW significantly reduced hepatic inflammation and stimulated extracellular matrix remodeling in experimental liver fibrosis.

## 1. Introduction

Liver fibrosis is characterized by the excessive production and deposition of extracellular matrix (ECM) proteins, such as collagen, and its occurrence inevitably coexists with a sustained inflammatory response [1,2]. Advanced liver fibrosis may result in cirrhosis and ultimately in liver failure and death [1]. Cirrhosis accounts for 4% of all deaths worldwide [3], and there are no specific anti-fibrotic therapeutic options available in clinic yet [4]. The pathophysiology of liver fibrosis involves the crosstalk of several parenchymal and nonparenchymal cells, including hepatocytes, hepatic stellate cells (HSCs), Kupffer cells (KCs), and liver sinusoidal endothelial cells (LSECs). Throughout the last decade, diverse anti-fibrotic pharmacological strategies have been proposed to inhibit the proliferation and activation of HSCs, to reduce the production and the deposition of the ECM, to reduce inflammation, or to promote liver protection [5,6]. However, these therapeutic approaches have shown limited efficacy and considerable side effects. Recently, different novel nanoscale therapeutic strategies have been suggested to treat liver fibrosis, overcoming the limitations of conventional pharmacological therapies by either protecting the liver from inflammation and oxidative stress or by targeting and treating macrophages [7,8].

Macrophages play essential roles during all stages of chronic liver disease, including fibrosis initiation, progression, and resolution [9,10]. In the initial phase, hepatocyte injury activates KCs, which secrete chemokines, such as CCL_2_, to attract monocytes from the bloodstream to the liver. During fibrosis progression, monocytes recruited to the liver differentiate into macrophages and secrete an array of pro-inflammatory and pro-fibrogenic factors that activate HSCs, which produce collagen and other fibers to restrict tissue damage propagation. Macrophages also contribute to fibrosis resolution mainly through the secretion of metalloproteinases and the stimulation of an anti-inflammatory and regenerative response [10]. The role of macrophages on fibrosis resolution may be dysregulated due to iterative and prolonged inflammatory stimuli occurring in chronic liver disease [2]. This hepatic milieu stimulates an uncontrolled production of inflammatory mediators by macrophages. This results in a defective formation of anti-inflammatory macrophages and an unpaired interplay between macrophages and HSCs, hepatocytes, or LSECs that may impair physiological liver regeneration [11,12]. Macrophages with anti-inflammatory, anti-fibrogenic, and pro-resolving capabilities are essential to restore physiological liver functions and homeostasis. For this reason, macrophage polarization to an anti-inflammatory phenotype has emerged as a potential therapeutic strategy to treat chronic liver disease [13,14].

Graphene nanostars linked to polyamidoamine (PAMAM) dendrimers have demonstrated excellent efficiency to target and treat macrophages with gene therapy in liver fibrosis [7]. Graphene nanostars (GNS) are formed by clusters of graphene-based single-walled carbon nanohorns (SWCNH) [15]. SWCNHs are nanostructures with a diameter of 2–5 nm and a length of 40–50 nm. SWCNHs aggregate to form spherical nanostars of around 100 nm in diameter [7,15,16]. PAMAM dendrimers are highly ordered and hyperbranched polymeric nanostructures formed by an ethylenediamine core, a repetitive branching amidoamine internal structure, and a primary amine terminal surface, which is easily modifiable to bind either peptides, nucleic acids, or other molecules [17].

Peroxisome proliferator-activated receptor γ (PPARγ) activation in macrophages has traditionally been associated with an anti-inflammatory phenotype [18]. PPARγ is a ligand-activated transcription factor included in the superfamily of nuclear receptors [19]. It has pleiotropic cellular effects, including lipid and glucose metabolism, adipocyte differentiation, cell growth control, and inflammation [19]. PPARγ heterodimerizes with the retinoid X receptor (RXR), and the PPARγ–RXR complex translocates to the cell nucleus to recruit diverse gene expression co-activators or co-repressors. The complex binds to DNA binding sequences and regulates the expression of target genes mainly related to inflammation and lipid and glucose metabolism [20]. In the absence of PPARγ activity, macrophages secrete high levels of pro-inflammatory cytokines and reduce the gene expression of anti-inflammatory mediators [18]. PPARγ activation and interaction with other signaling pathways, such as NF-kB and JAK2/STAT1, regulates macrophage polarization [21] and induces a phenotypic change from pro-inflammatory M1 to anti-inflammatory M2 macrophages [22]. In this context, diverse PPARγ agonists have been proposed to induce an anti-inflammatory response [23]. GW1929 is a potent tyrosine-based non-thiazolidinedione PPARγ agonist [19] with a well-established efficacy for macrophage M2 polarization [22,24,25,26]. However, the use of most PPARγ full agonists is greatly restricted in conventional therapy due to their wide and severe side effects, which include increased cardiovascular risk, bone loss, edema, and fluid retention [27,28,29]. Therefore, an ideal nanoscale delivery system for GW1929 should selectively target macrophages and transport a reduced drug dose to avoid possible side effects and to overcome the limitations of conventional dosage forms. Here, we sought to selectively activate PPARγ in liver macrophages with a low dose of the GW1929 agonist linked to dendrimer–graphene nanostars to selectively stimulate a M2 anti-inflammatory macrophage phenotype and to boost macrophage-driven liver fibrosis resolution.

## 2. Materials and Methods

### 2.1. Synthesis of Dendrimer–Graphene Nanostars Linked to GW1929 (DGNS-GW) or Mannitol (DGNS-Man)

Carbon graphene oxide nanohorns, GW1929 PPARγ agonists, and mannitol were supplied by Sigma (St. Louis, MO, USA). Generation 5 (G5) poly (amidoamine) (PAMAM) dendrimer was purchased from Dendritech Inc. (Midland, MI, USA). Oxidized GNS were dispersed in dimethyl sulfoxide (DMSO) (500 μg/mL) and separated via the incubation of the dispersion in an ultrasound bath (Selecta, Barcelona, Spain) at a frequency of 50 kHz and potency 360 W for 15 min as previously described [7]. A total of 100 μL of carbon nanohorns was mixed with 900 μL of 1 mg/mL of free access crosslinking agents 1-Ethyl-3-(3-dimethylaminopropyl)-carbodiimide (EDC)/N-Hydroxysuccinimide (NHS) 1:1 with 30 μL of PAMAM dendrimer 25% *v*/*v*. GNS, EDC/NHS, and G5 PAMAM were incubated for 2 h in the ultrasound bath to have the nanohorns separated for the reaction, with constant temperature at 25 ± 2 °C with ice. Dispersions were centrifuged at 21,000 Gs for 10 min and washed three times with DMSO. A total of 200 μL of DMSO-dispersed DGNS (50 μg/mL) were mixed with 800 μL of DMSO; 10, 2, 1, or 0.5 mg of DMSO-soluble GW1929 or mannitol; and 1 mg/mL of EDC/NHS for a second reaction with crosslinking agents in constant agitation in a magnetic stirrer at 25 °C for two hours. Dispersions were centrifuged at 21,000 Gs for 10 min and washed one time with DMSO and four times with phosphate-buffered saline (PBS) for further analysis. An adequate GW1929 concentration for maintaining a negative nanoparticle surface was established using the variations in Zeta potential from positive (DGNS) to negative (DGNS-GW or DGNS-Man).

### 2.2. Physicochemical Characterization of Nanoparticles

Nanoparticle hydrodynamic size properties were determined by Dynamic Light Scattering (DLS), using a Zetasizer nano ZS (Malvern Instruments Ltd., Worcestershire, UK). Size measurements were carried out at 25 °C and at fixed angle of 173° by analyzing the intensity of the scattered light supplied by a helium–neon laser (maximum output power = 4 mW, beam wavelength = 633 nm). DLS data were calculated from the autocorrelation function of scattered light by means of two mathematical methods—the method of cumulants and Dispersion Technology Software nano v. 5.10 (Malvern Instruments Ltd.). Two important parameters were obtained through the cumulants analysis: the mean of particle hydrodynamic diameter (Z-average) and the width of the particle size distribution (polydispersity index—PDI). Samples for measurements were prepared as follows: 50 μL of GNS, DGNS, DGNS-GW, and DGNS-Man suspension was dispersed in 950 μL of PBS in an ordinary cuvette. The reported values of Z-Average and PDI corresponded to the average of approximately 40 measurement runs from three different dispersions. A total of 50 μL of GNS, DGNS, DGNS-GW, and DGNS-Man dispersed in 950 μL of PBS was used to perform Zeta-potential measurements using disposable folded capillary cells (DTS1070, Malvern Instruments Ltd., Worcestershire, UK) in a Zetasizer nano ZS.

### 2.3. Cell Culture

Mouse RAW 264.7 macrophages (ATCC, Manassas, VA, USA) were cultured with Dulbecco’s modified Eagle’s medium (DMEM) supplemented with 10% fetal bovine serum (FBS) and 50 U/mL penicillin/streptomycin. Cells were grown at 37 °C and 5% CO_2_ in a water-jacketed incubator. For macrophage polarization and nanoparticle uptake experiment, cells were seeded in six-well plates at a density of 2 × 10^4^ cell/cm^2^ supplemented with low FBS (1%). At 16 h after seeding, cells were treated with or without DGNS-GW or DGNS-Man (100 ng/mL) and with TNF-α (5 ng/mL, Life Technologies, Carlsbad, CA, USA) for three days with the daily renewal of culture media. After three days, cells were harvested with a 1 mL of TRIZOL reagent (Gibco-Invitrogen, Paisley, UK) for RNA isolation. Nanoparticle uptake post-TNF-α stimulation and DGNS-GW treatment was determined using black aggregate quantification. Black aggregates of DGNS-GW were visualized at high magnification to establish the number of cells incorporating the nanostars. The percentage of cells incorporating DGNS-GW was calculated as follows: the number of cells with black aggregates/total number of cells per field × 100. At least 30 different fields were used to calculate the uptake percentage per condition.

### 2.4. Animal Studies

Male Balb/c mice were purchased from Charles River Laboratories (Charles River, Saint Aubin les Elseuf, France). The study was performed according to the criteria of the Investigation and Ethics Committees of the Hospital Clínic Universitari of Barcelona. Animals were maintained in a temperature-controlled room (22 °C) on a 12 h light–dark cycle. After arrival, mice were continuously fed ad libitum until euthanasia (endpoint). For liver fibrosis induction, mice were injected with intraperitoneal CCl_4_ diluted 1:8 *v*/*v* in corn oil twice a week for 10 weeks. After fibrosis induction, dispersions of DGNS-Man or DGNS-GW in PBS were intravenously injected (50 μg/Kg DGNS and 2.5 mg/Kg GW1929 or mannitol) every 3 days for 10 days (4 injections in total). Animals were euthanized the day after the last intravenous injection (at day 11). Liver samples and serum were collected and frozen for further analysis. Serum alanine aminotransferase (ALT), aspartate aminotransferase (AST), albumin and total protein were measured using a BS-200E Chemistry Analyzer (Mindray Medical international Ltd., Shenzhen, China). Liver weight/body weight ratio was calculated as follows: liver weight/body weight × 100 g.

### 2.5. Gene Expression Assay

Total RNA from liver was extracted using commercially available RNeasy RNA extraction kit (Qiagen, Germantown, MD, USA). RNA from cells was extracted using TRIzol^TM^ kit (Gibco-Invitrogen, Paisley, UK). A 1 μg aliquot of total RNA was reverse transcribed using a complementary DNA synthesis kit following the manufacturer’s instructions (High-Capacity cDNA Reverse Transcription Kit, Applied Biosystems, Foster City, CA, USA). Gene expression assays were designed using the Taqman Gene Expression assay software (Applied Biosystems). Probes and primers for gene expression assays (Applied Biosystems) were selected as follows: IL-10 (Taqman assay reference from Applied Biosystems: Mm00439614_m1), NOS2 (Mm00440502_m1), COX-2 (Mm00478374_m1), MRC1 (Mm00485148_m1), ARG-1 (Mm00475988_m1), Col1A1 (Mm00801666_g1), α-SMA (Mm01204962_gH), TIMP-1 (Mm01341360_g1), MMP-9 Mm00442991_m1), TIMP-2 (Mm00442991_m1), MMP-2 (Mm00439498_m1), HGF (Mm01135184_m1), IGF-1 (Mm00439560_m1), VEGF (Mm00437306_m1), and hypoxanthine phosphoribosyltransferase (HPRT) (Mm03024075_m1) used as an endogenous standard. Real-time quantitative PCR was analyzed in duplicate and performed with a Lightcycler-480 II (Roche Diagnostics). For each PCR reaction, a 10 μL aliquot of the total volume reaction of Taqman probes and primers, the FastStart TaqMan Master (Applied Biosystems), and 1:8 diluted complementary DNA were used. The TaqMan probe fluorescence signal was captured during each of the 45 cycles (denaturing 10 s at 95 °C, annealing 15 s at 60 °C, and extending 20 s at 72 °C). The relative gene expression was quantified using the comparative threshold cycle (CT), which was inversely related to the abundance of mRNA transcripts in the initial sample. The mean CT of the duplicate measurements was used to calculate ΔCT (difference in CT between the target and endogenous standard gene for each sample). ΔΔCT was obtained from the normalization of ΔCT values per each sample with the mean ΔCT of control samples. The relative expression of a gene was expressed as the fold induction of the target gene compared with the control primers, according to the formula 2^−ΔΔCT^.

### 2.6. Fibrosis Quantification

For fibrosis quantification, the liver was excised, washed with PBS, and fixed with 10% buffered formaldehyde solution for 24 h. Afterwards, the liver tissue was embedded in paraffin and 6 μm liver sections were obtained. Before staining, paraffin was removed using xylene, xylene/ethanol 1:1, ethanol, ethanol/deionized water 1:1, and deionized water (5 min in each solution). Liver sections were stained in 0.1% Sirius Red F3B (Sigma) with saturated picric acid (Sigma). The relative fibrosis area (expressed as a percentage of total liver area) was analysed in 10 fields of Sirius red-stained liver sections per animal using the morphometry software ImageJ. To evaluate the relative fibrosis area, the measured collagen area was divided by the net field total liver area and then multiplied by 100. From each animal analysed, the percentage of fibrosis area was calculated and the average value presented.

### 2.7. Immunofluorescence and Imaging in Liver Tissues

For proliferating cell nuclear antigen (PCNA), pro-inflammatory M1-like marker (nitric oxide synthase 2: NOS2, cyclooxygenase-2: COX-2), and anti-inflammatory M2-like marker (mannose receptor 1: MRC1, arginase 1: ARG1) immunostaining, the liver was excised, washed with PBS, and fixed with 10% buffered formaldehyde solution for 24 h. Afterwards, the liver tissue was cryo-protected with 30% sucrose solution (in PBS) for another 24 h, embedded using Tissue-Tek OCT compound (Sakura Fineteck USA, Torrance, CA, USA), and frozen. For immunostaining, 6 μm liver sections were obtained using a cryostat (Leica Biosystems, Wetzlar, Germany). Liver sections underwent 1% SDS solution antigen retrieval for 5 min at room temperature and then were blocked with 5% normal goat serum in PBS for another hour. Liver sections were incubated with rabbit polyclonal anti-PCNA antibody (1:100, Abcam, Cambridge, MA, USA), rabbit anti-NOS2 polyclonal antibody (1:100, Thermofisher Scientific, Waltham, MA, USA), rabbit anti-COX-2 polyclonal antibody (1:100, Proteintech), rabbit anti-ARG1 polyclonal (1:100, Thermofisher Scientific), or rabbit polyclonal anti-MRC1 (1:100, Abcam, Cambridge, MA, USA) for 16 h at 4 °C. Primary antibodies were revealed using donkey-anti-rabbit IgG Alexa Fluor 594 (1:500, Jackson ImmunoResearch Laboratories, West Grove, PA, USA) or Cy3-conjugated donkey-anti-rabbit IgG (1:500, Jackson ImmunoResearch Laboratories, West Grove, PA, USA) incubated for 2 h at room temperature. The presence of PCNA, COX-2, NOS2, ARG1, and MRC1 was visualized with an epifluorescence microscope. DAPI (Vectashield, Vector laboratories, Burlingame, CA, USA) was used to counterstain cell nuclei. The percentage of positive PCNA cells was calculated as follows: PCNA positive nuclei/total number of cells defined by DAPI nuclei per field × 100. PCNA positive cells were analysed in 10 fields per animal and the average values are presented.

### 2.8. Statistical Analysis

All data were expressed as mean ± standard error of mean (S.E.M). The number of replicates per each experiment is detailed in figure legends. The statistical analysis of the results was performed through Student’s *t*-tests with GraphPad Prism v6.0a. Differences were considered statistically significant when the *p*-value ≤ 0.05.

## 3. Results

### 3.1. Synthesis and Physicochemical Characterization of Dendrimer–Graphene Nanostars Linked to GW1929 PPARγ Agonist

**Figure 1:**
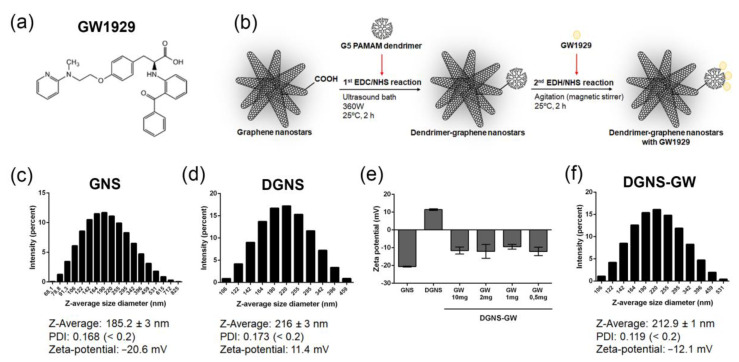
Synthesis and physicochemical characterization of dendrimer–graphene nanostars linked to the GW1929 agonist. (**a**) Chemical structure of GW1929. (**b**) Schematic representation of the chemical synthesis process of dendrimer–graphene nanostars linked to GW1929. (**c**) Representative particle hydrodynamic size histogram of graphene nanostars (GNS) obtained through dynamic light scattering (DLS) showing the values of the Z-average, polydispersity index (PDI), and Zeta-potential. (**d**) Representative particle hydrodynamic size histogram of dendrimer–graphene nanostars (DGNS) obtained via DLS showing the Z-average, PDI, and Zeta-potential values. (**e**) Zeta-potential of GNS, DGNS, and DGNS dispersed in PBS linked to different quantities of GW1929 (10 mg, 2 mg, 1 mg, and 0.5 mg) per milliliter of DGNS suspension. (**f**) Representative particle hydrodynamic size histogram of DGNS linked to GW1929 (DGNS-GW) obtained via DLS showing the Z-average, PDI, and Zeta-potential values. N = 3 different measurements. For (**e**), data are shown as mean ± S.E.M.

We used a synthesis method modified from a previous design of dendrimer–graphene nanostars (DGNS) [7] to obtain DGNS linked to a low dose of the GW1929 PPARγ agonist (DGNS-GW) to induce macrophage M2 polarization for the treatment of liver fibrosis. GW1929 (Figure 1a) has a carboxylic group on its chemical structure that can react with the primary amines in G5 PAMAM dendrimers in the presence of EDC/NHS crosslinking agents. DGNS-GW were synthetized in two consecutive chemical reactions. First, the crosslinking agents EDC/NHS and G5 PAMAM dendrimers were incubated with carboxylated GNS using continuous ultrasonic agitation for two hours at a constant temperature of 25 °C. Then, GW1929 was covalently linked to DGNS through a second reaction with EDC/NHS under constant magnetic stirring and temperature (25 °C) for two hours in order to obtain DGNS-GW (Figure 1b).

The measurements of the hydrodynamic diameter via DLS revealed a Z-average of 185.2 ± 3 nm in carboxylated GNS and a negative Zeta-potential (−20.6 mV) due to the presence of carboxylic groups (Figure 1c). The Z-average rose to 216 ± 3 nm when PAMAM dendrimers were covalently incorporated (Figure 1d). The Zeta-potential of DGNS switched to positive (11.4 mV), resulting in a hyperosmotic nanoparticle dispersion (Figure 1d). Different concentrations of GW1929 were incubated with DGNS (10 µg/mL) to determine the minimum drug quantity required to obtain biologically compatible nanostars with a negative Zeta-potential surface. DGNS switched to negative Zeta-potential when they were linked with 10 mg of GW1929 (Figure 1e). DGNS demonstrated similar negative Zeta-potential when incubated with 20-fold less of the free drug (0.5 mg) (Figure 1e). We used this formulation with a low drug levels of GW1929 linked to DGNS for subsequent experiments. DLS measurements revealed a Z-average size of DGNS-GW of 212.9 ± 1 nm, indicating no significant change in the hydrodynamic diameter of drug-linked particles compared to DGNS, and a Zeta-potential of −12.1 mV (Figure 1f). All GNS, DGNS, and DGNS-GW preparations demonstrated a uniform particle size distribution and a low polydispersity index (PDI < 0.2) (Figure 1c,d,f).

### 3.2. In Vitro Evaluation of the Activity of DGNS-GW to Stimulate Macrophage Polarization

**Figure 2:**
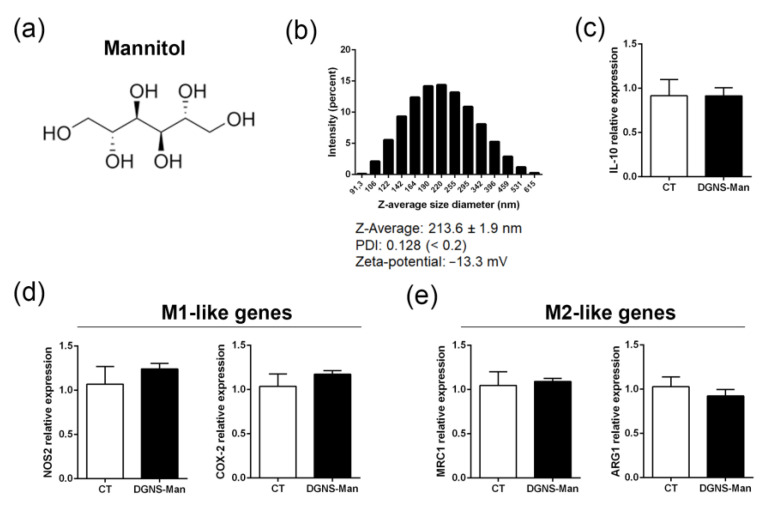
Physicochemical characterization and in vitro validation of dendrimer–graphene nanostars linked to mannitol as control nanoparticles. (**a**) Chemical structure of mannitol. (**b**) Representative particle hydrodynamic size histogram of dendrimer–graphene nanostars linked to mannitol (DGNS-Man) obtained via DLS, showing the values of Z-average, PDI, and Zeta-potential. (**c**) IL-10 expression in control mouse RAW 264.7 macrophages and macrophages stimulated with DGNS-Man for three days. (**d**) M1-like gene expression (NOS2 and COX-2) in control mouse RAW 264.7 macrophages and macrophages stimulated with DGNS-Man for three days. (**e**) M2-like gene expression (MRC1 and ARG1) in control mouse RAW 264.7 macrophages and macrophages stimulated with DGNS-Man for three days. For (**b**), N = 3 different measurements. For (**c**–**e**), experiments were performed in sextuplicate. Data are shown as mean ± S.E.M. No significant differences were observed using Student’s *t*-test.

We then investigated the potential of DGNS-GW in macrophage polarization in vitro. We first synthetized DGNS linked to mannitol (DNGS-Man) (Figure 2a) as control nanoparticles. Mannitol has previously been used as a standard control in macrophage polarization experiments [30,31,32]. DLS measurements revealed a Z-average of DGNS-Man of 213.6 ± 1.9 nm, a uniform nanoparticle size distribution, and a low PDI (Figure 2b). DGNS-Man also presented a Zeta-potential of −13.3 mV (Figure 2b), thus demonstrating no significant differences in terms of nanoparticle characteristics as compared to DGNS-GW. To confirm that the treatment with DGNS-Man had no impact on PPARγ activation, we measured the expression of the downstream PPARγ target interleukin 10 (IL-10) in mouse RAW 264.7 macrophages treated with DGNS-Man. We found no differences in IL-10 expression in macrophages treated with DGNS-Man compared to control macrophages without any stimulation (Figure 1c). To ensure that mannitol was not exerting any effect on macrophage polarization, we evaluated the expression of pro-inflammatory M1-like genes (nitric oxide synthase 2, NOS2; cyclooxygenase-2, COX-2) and anti-inflammatory M2-like genes (mannose receptor 1, MRC1; arginase 1, ARG1) in macrophages treated with DGNS-Man. We found no differences in the M1-like gene expression (Figure 2d) or M2-like gene expression (Figure 2e) in macrophages treated with DGNS-Man compared to non-stimulated macrophages, indicating the suitability of DGNS-Man as control nanoparticles for further experiments.

**Figure 3:**
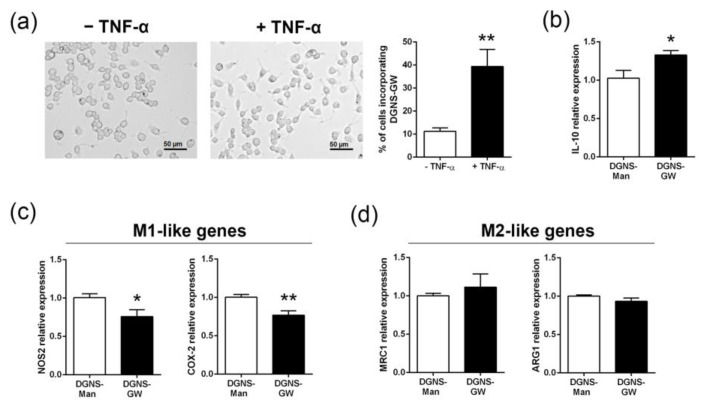
In vitro nanoparticle uptake and macrophage polarization following the treatment with dendrimer–graphene nanostars linked to GW1929. (**a**) Uptake experiment using RAW 264.7 macrophages incubated with dendrimer–graphene nanostars linked to GW1929 (DGNS-GW) for 3 days in the presence or absence of TNF-α (5 ng/mL), showing representative images and percentages of cells incorporating nanoparticles. (**b**) IL-10 expression in mouse RAW 264.7 macrophages in the presence of TNF-α and treated with dendrimer–graphene nanostars linked to mannitol or GW1929 for three days. (**c**) M1-like gene expression (NOS2 and COX-2) in mouse RAW 264.7 macrophages in the presence of TNF-α and treated with dendrimer–graphene nanostars linked to mannitol or GW1929 for three days. (**d**) M2-like gene expression (MRC1 and ARG1) in mouse RAW 264.7 macrophages in the presence of TNF-α and treated with dendrimer–graphene nanostars linked to mannitol or GW1929 for three days. Experiments were performed in sextuplicate. Data are shown as mean ± S.E.M. * indicates *p* < 0.05 and ** indicates *p* < 0.01 using Student’s *t*-test.

To investigate whether DGNS could be incorporated by macrophages and retained for long periods, we incubated macrophages with DGNS-GW for three days with or without TNF-α inflammatory stimulus. Approximately 40% of macrophages still conserved DGNS-GW after three days of treatment under TNF-α stimulation. In contrast, only 10% of non-stimulated macrophages conserved DGNS-GW after three days of nanoparticle treatment (Figure 3a). These results reinforce the fact that DGNS-GW could be selectively incorporated and retained by pro-inflammatory macrophages in livers undergoing chronic inflammation, where they could act as drug delivery systems for efficient macrophage-targeted nanotherapeutics.

We then investigated the pharmacological effectivity of DGNS-GW on the activation of PPARγ downstream signals via the evaluation of IL-10 gene expression. IL-10 expression was higher in mouse macrophages stimulated in vitro with TNF-α and treated with DGNS-GW compared to macrophages treated with DGNS-Man stimulated with TNF-α (Figure 3b). Moreover, the expression of M1-like genes (NOS2 and COX-2) decreased in macrophages treated with DGNS-GW compared to macrophages treated with DGNS-Man (Figure 3c), without affecting the expression of M2-like genes (MRC1 and ARG1) (Figure 3d).

### 3.3. Evaluation of the Therapeutic Utility of DGNS-GW in Mice with Liver Fibrosis

We then evaluated the therapeutic utility of DGNS-GW in a mouse model of liver fibrosis. The conventional dosage form of the systemic treatment of GW1929 is between 5 mg/kg and 20 mg/kg in mice [33,34,35]. We wondered whether the selective activation of PPARγ in liver macrophages with a low dose (1/4 of conventional dose) of GW1929 that was linked to DGNS could be effective in stimulating a selective M2 anti-inflammatory macrophage phenotype and boosting macrophage-driven liver fibrosis resolution.

**Figure 4:**
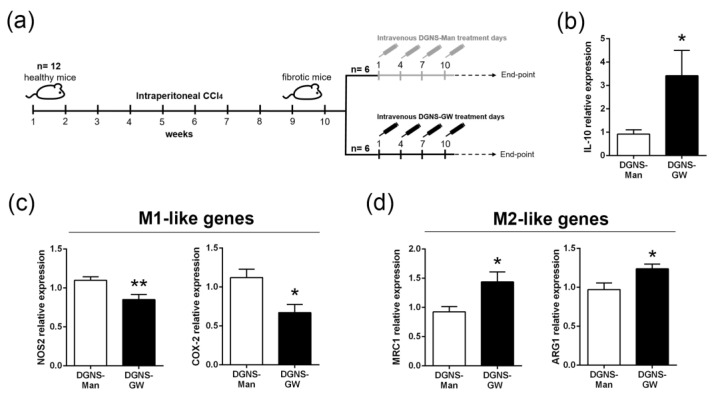
Hepatic immunomodulation of macrophage gene expression profile in mice with liver fibrosis treated with DGNS-GW. (**a**) Schematic illustration indicating the time points of fibrosis induction with CCl_4_ and the administration schedule of dendrimer–graphene nanostars linked to mannitol (DGNS-Man) or GW1929 (DGNS-GW). (**b**) IL-10 expression in the livers of fibrotic mice treated with DGNS-Man or DGNS-GW. (**c**) M1-like gene expression (NOS2 and COX-2) in the livers of fibrotic mice treated with DGNS-Man or DGNS-GW. (**d**) M2-like gene expression (MRC1 and ARG1) in the livers of fibrotic mice treated with DGNS-Man or DGNS-GW. N = six mice per group. Data are shown as mean ± S.E.M. * indicates *p* < 0.05 and ** indicates *p* < 0.01 using Student’s *t*-test.

Liver fibrosis was induced in twelve male Balb/c mice via intraperitoneal injections of the hepatotoxic molecule CCl_4_ twice a week for 10 weeks. We intravenously administered DGNS-GW or DGNS-Man every 3 days for 10 days (four injections in total) to fibrotic mice (Figure 4a). To ensure that DGNS-GW with low drug dose levels were efficient for PPARγ signaling activation, we quantified IL-10 gene expression in the liver of fibrotic mice treated with DGNS-GW. The IL-10 expression was approximately four times higher in the liver of fibrotic mice treated with DGNS-GW compared to mice treated with DGNS-Man (Figure 4b). These results indicated that DGNS could be an adequate nanoscale delivery system for transporting a low dose of GW1929 agonist to macrophages in order to overcome the side effects and limitations of conventional dosages and formulations.

In accordance with our in vitro results, the selective activation of PPARγ in macrophages from fibrotic livers reduced the expression and synthesis of M1-like pro-inflammatory genes (NOS2 and COX-2) in the liver of fibrotic animals (Figure 4c and Figure S1). Moreover, treatment with DGNS-GW increased the expression and synthesis of anti-inflammatory M2-like genes (MRC1 and ARG1) in the liver of fibrotic mice treated with DGNS-GW compared to DGNS-Man (Figure 4d and Figure S1). These results suggest that the selective activation of PPARγ in hepatic macrophages with DGNS-GW could exert hepatic immunomodulatory activity via the re-education of macrophages on an anti-inflammatory phenotype.

**Figure 5:**
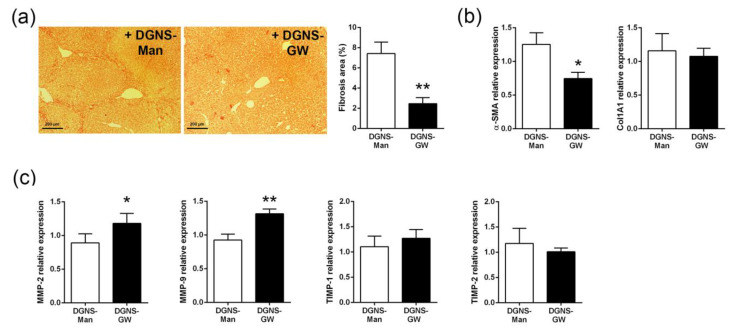
Effect of macrophage-targeted DGNS-GW therapy on liver fibrosis and the expression of extracellular matrix-related genes. (**a**) Representative images and quantification of Sirius Red staining in the livers of fibrotic mice treated with dendrimer–graphene nanostars linked to mannitol (DGNS-Man) or GW1929 (DGNS-GW). (**b**) Alpha smooth muscle actin (α-SMA) and collagen I (Col1A1) expression in the livers of fibrotic mice treated with DGNS-Man or DGNS-GW. (**c**) Tissue inhibitor metalloproteinases (TIMP-1 and TIMP-2) and gelatinase metalloproteinases (MMP-9 and MMP-2) expression in the livers of fibrotic mice treated with DGNS-Man or DGNS-GW. N = six mice per group. Data are shown as mean ± S.E.M. * indicates *p* < 0.05 and ** indicates *p* < 0.01 using Student’s *t*-test.

Since macrophages play essential roles in the balance between fibrogenesis and regression and PPARγ agonists have been proven to reduce experimental liver fibrosis [23,36,37], we then evaluated the potential therapeutic utility of macrophage-targeted DGNS-GW in ECM remodeling in liver fibrosis. We stained collagen fibers in the livers of fibrotic mice treated with DGNS-GW or DGNS-Man. Fibrotic mice treated with DGNS-GW showed approximately 60% less fibrotic area compared to mice treated with DGNS-Man, illustrated through Sirius Red staining (Figure 5a). This reduction in liver fibrosis did not affect the serum markers of liver damage (ALT and AST) or serum markers of hepatic function (albumin and total protein) (Table S1). The reduction in liver fibrosis was associated with a decrease in the liver expression of alpha smooth muscle actin (α-SMA), without altering the expression of collagen 1 (Figure 5b). Since macrophages modulate hepatic fibrosis regression through the secretion of matrix metalloproteinases (MMPs) [10,14] but activated HSCs produce tissue inhibitor metalloproteinases (TIMPs) suppressing MMPs activity in late stages of liver fibrosis [2], we wondered whether the selective activation of PPARγ in hepatic macrophages could modulate the liver expression of MMPs and TIMPs. The treatment with DGNS-GW in fibrotic mice increased the expression of the gelatinases MMP-2 and MMP-9 but did not affect the hepatic expression of the associated TIMPs (TIMP-1 and TIMP-2) (Figure 5c), suggesting that the selective activation of PPARγ in hepatic macrophages with DGNS-GW impairs liver fibrosis and modulates macrophage fate towards a pro-resolutive phenotype via the induction of the expression of these extracellular matrix metalloproteinases.

**Figure 6:**
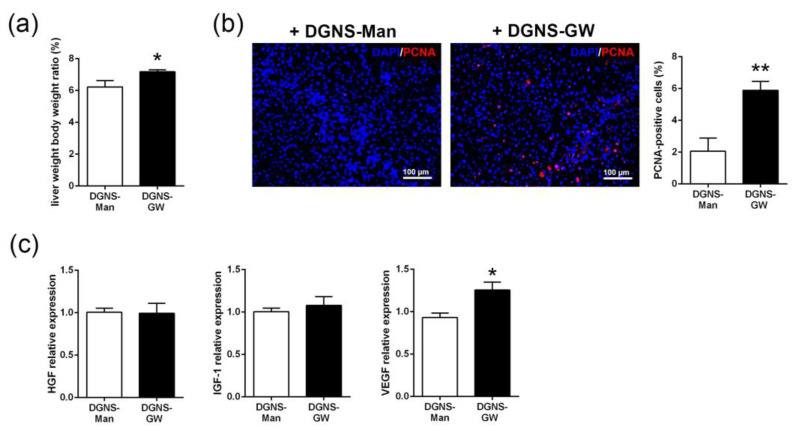
Effect of macrophage-targeted DGNS-GW therapy on liver regeneration. (**a**) Liver restoration rate in fibrotic mice treated with dendrimer–graphene nanostars linked to mannitol (DGNS-Man) or GW1929 (DGNS-GW). (**b**) Proliferating cell nuclear antigen (PCNA) immunofluorescence staining representative images and quantification (percentage of PCNA positive cells) in the livers of fibrotic mice treated with DGNS-Man or DGNS-GW. (**c**) Hepatocyte growth factor (HGF), Insulin-like growth factor-1 (IGF-1), and vascular endothelial growth factor (VEGF) expression in the livers of fibrotic mice treated with DGNS-Man or DGNS-GW. N = six mice per group. Data are shown as mean ± S.E.M. * indicates *p* < 0.05 and ** indicates *p* < 0.01 using Student’s *t*-test.

Fibrotic mice treated with DGNS-GW displayed a significant increase in liver mass compared to fibrotic mice treated with DGNS-Man (Figure 6a). We speculated whether the selective activation of PPARγ in hepatic macrophages could favor hepatic regeneration in the context of liver fibrosis. We evaluated the abundance of the proliferating cellular nuclear antigen (PCNA) by immunohistochemistry in the livers of fibrotic mice treated with DGNS-Man or DGNS-GW. Fibrotic mice treated with DGNS-GW displayed an increase in the number of PCNA-positive cells, indicating an augmented hepatic cellular proliferation (Figure 6b). Then, we evaluated the hepatic expression of hepatocyte growth factor (HGF), insulin-like growth factor-1 (IGF-1), and vascular endothelial growth factor (VEGF), since the pro-resolutive macrophage phenotype is characterized by the secretion of these growth factors, which promote liver cell proliferation and blood vessel development [12]. We did not find significant differences in the hepatic expression of HGF and IGF-1 (Figure 6c). In contrast, fibrotic mice treated with DGNS-GW showed a significant increase in the hepatic expression of VEGF compared to fibrotic mice treated with DGNS-Man (Figure 6c), indicating that PPARγ-activated liver macrophages stimulate hepatic proliferation in part via the synthesis of VEGF.

## 4. Discussion

PPARγ agonists have traditionally been used for the treatment of diabetes mellitus and other metabolic disorders [38]. Diverse PPARγ full agonists have also been proposed to stimulate macrophage anti-inflammatory responses [22,23]. However, the use of full agonists in biological systems is greatly limited due to side effects [27,28,29]. Some polymeric nanoparticles have already been suggested for the delivery of low dose PPARγ agonists to overcome toxicity-related limitations [39,40,41]. Here, we sought to design a macrophage-selective treatment with a low dose of GW1929 PPARγ agonist linked to carbon-based nanoparticles (DGNS-GW) as drug delivery systems for the treatment of liver fibrosis.

We first characterized nanoparticle surface charge and hydrodynamic diameter via dynamic light scattering. Cationic carbon nanoparticles (with positive surface charge) have been associated with toxicity in macrophages and cells from epithelial origin [42]. Chemical linkage between GW1929 and DGNS solved this potential biocompatibility problem, exhibiting a negative surface charge. Indeed, we have previously demonstrated that anionic pDNA-DGNS presented no harmful effects on human endothelial cells, which are the primary cells in blood vessels and the first biological barrier for intravenously administered formulations [7]. The hydrodynamic size of DGNS-GW resulted in 212.9 nm. Since most nanoparticles administered in vivo that are over 200 nm are supposed to be primarily incorporated by macrophages [13,43], DGNS appear as a suitable nanoscale system for GW1929 agonist delivery to macrophages. Indeed, macrophages are more efficient in incorporating functionalized anionic DGNS under TNF-α inflammatory stimulation [7]. In a previous report, approximately 80% of macrophages incorporated these nanoparticles as fast as three hours after TNF-α stimulation and only 20% of macrophages without TNF-α stimulation were able to engulf them [7]. Here, we observed that 40% of macrophages still conserved DGNS-GW after three days of treatment under TNF-α stimulation. We also synthetized and characterized DGNS linked to mannitol as control nanoparticles for subsequent in vitro and in vivo experiments. Mannitol has previously been used as a standard control in macrophage polarization experiments [30,31,32]. We further confirmed that DGNS-Man had no impact on macrophage polarization. Both DGNS linked to mannitol or GW1929 presented no significant differences according to nanoparticle hydrodynamic size and Zeta-potential, illustrating the suitability of DGNS-Man as control nanoparticles for our study.

Liver fibrosis is associated with a sustained inflammatory milieu [1,2]. TNF-α is a prominent cytokine driving inflammation in chronic liver disease [44,45]. Moreover, TNF-α has been associated with the inhibition of PPARγ both at pre-translational and post-translational levels [46]. Heming et al. demonstrated that macrophages display sustained immune responses in the absence of PPARγ signaling, impairing their ability to re-program towards a pro-resolving phenotype [47]. Since PPARγ activation has been linked to macrophage anti-inflammatory phenotypes [18], we tested the functional ability of DGNS-GW on macrophage polarization in cells under TNF-α stimulation and in the liver of mice with liver fibrosis induced by the i.p. administration of CCl_4_ as an in vivo inflammatory niche. The chronic administration of CCl_4_ has been classically used to promote chronic liver injury in animal models. CCl_4_ induces the formation of hepatic regenerative nodules surrounded by fibrotic tracts and the infiltration of pro-inflammatory macrophages that sustain the inflammatory response [48]. We have previously demonstrated the selectivity of anionic DGNS linked to plasmids to target and treat inflammatory macrophages with gene therapy in the liver of fibrotic mice [7]. Here, livers of fibrotic mice treated with anionic DGNS-GW displayed a reduction in the gene and protein expression of M1 pro-inflammatory factors. PPARγ activation has been linked to the inhibition of the molecular signaling of the nuclear factor NF-kB [49], which can ultimately result in the downregulation of pro-inflammatory genes. This fact may explain the observed effects on the decreased expression of COX-2 and NOS2 in both in vitro and in vivo experiments under a constant inflammatory stimulus.

The expression of M2 anti-inflammatory genes and proteins increased in the livers of fibrotic mice following macrophage-selective PPARγ activation with DGNS-GW. Anti-inflammatory M2 polarization has been classically associated with the activation of macrophages with IL-4 and IL-13 interleukin signals [50]. The anti-inflammatory IL-4 or IL-13 initiates a cytoplasmic signaling cascade that culminates in the activation of STAT6 transcription factor [50]. Phosphorylated STAT6 dimerizes and translocates to the nucleus to induce the expression of its target genes, including M2 macrophage markers (MRC1 and ARG1) and other regulators of PPARγ [50]. While the instructions for M2 macrophage polarization may not be directly linked to PPARγ activation, the acquisition and long-term maintenance of this phenotype requires PPARγ activity [50]. This may explain the upregulation of the expression of M2 anti-inflammatory genes observed in the livers of mice treated with DGNS-GW for ten days. Altogether, our results reinforce the fact that PPARγ acts as a nuclear regulator of inflammation in macrophages. In the context of chronic liver disease, the macrophage-selective activation of PPARγ may be a promising therapeutic strategy for promoting macrophage polarization from pro-inflammatory to anti-inflammatory phenotypes.

Since chronic liver inflammation and fibrosis are two phenomena that are tightly associated [51], we evaluated the anti-fibrotic utility of DGNS-GW on mice with liver fibrosis. The modulation of PPARγ has been proven to attenuate HSC activation and to reduce liver fibrosis [36]. Our results revealed a reduction in the liver fibrotic area and a decreased expression of liver α-SMA in mice treated with DGNS-GW. IL-10 expression is regulated by PPARγ and has been directly linked to α-SMA reduction [52]. Interestingly, we did not observe a significant reduction in collagen I and TIMPs gene expression following selective macrophage PPARγ activation with DGNS-GW. This fact illustrated that DGNS-GW treatment may not directly modulate HSC activity. Macrophages play an essential role in extracellular matrix remodeling through the secretion of MMPs [10]. Indeed, DGNS-GW treatment in fibrotic mice increased the expression of liver gelatinase MMPs (MMP-2 and MMP-9). Therefore, the anti-fibrotic effect of DGNS-GW treatment may be associated with the increase in macrophage MMPs secretion rather than the inhibition of HSC activity. We finally observed a significant hepatic regeneration and an increase in PCNA-positive cells along fibrotic tracts in the liver of fibrotic mice treated with DGNS-GW. This correlated with an augmented liver VEGF expression. PPARγ activation has been associated with VEGF production in macrophage cell lines [53]. Moreover, VEGF has been linked to fibrosis resolution through the stimulation of scar-associated macrophages [54]. Taken together, DGNS-GW treatment may induce liver macrophage VEGF secretion to stimulate the proliferation of pro-resolutive liver cells, such as scar-associated macrophages. However, we cannot exclude other cellular or molecular components involved in the anti-fibrotic effect of DGNS-GW treatment. Overall, our results indicate that macrophage-selective PPARγ activation with DGNS-GW may polarize liver macrophages towards a pro-resolutive phenotype to stimulate extracellular matrix remodeling in liver fibrosis.

## 5. Conclusions

We designed dendrimer–graphene nanostars linked to a low dose of the GW1929 PPARγ agonist (DGNS-GW) to induce a selective activation of PPARγ in macrophages in fibrotic liver. The treatment with DGNS-GW effectively activated PPARγ signaling in macrophages in in vitro and in vivo experiments, illustrated by the increase in IL-10 expression. DGNS-GW accumulated in macrophages stimulated with TNF-α and attenuated their pro-inflammatory phenotype. Accordingly, the treatment with DGNS-GW in fibrotic mice promoted a macrophage switch from pro-inflammatory M1 to anti-inflammatory M2 phenotypes. The reduction of hepatic inflammation correlated with a reduction in liver fibrosis and an increase in gelatinase MMPs (MMP-2 and MMP-9). Moreover, the treatment with DGNS-GW induced liver regeneration and augmented liver VEGF expression. In conclusion, the selective activation of PPARγ in hepatic macrophages using DGNS-GW reduces hepatic inflammation and fibrosis. This study gives new insights into the relationship between PPARγ activation in hepatic macrophages and fibrosis resolution and highlights that DGNS-GW is a promising macrophage-targeted nanoscale therapy for chronic liver disease.

## Data Availability

All data are available in the main manuscript text and Supplementary Material.

## Supplementary Materials

The following supporting information can be downloaded at: https://www.mdpi.com/article/10.3390/pharmaceutics15051452/s1, Table S1: Serum parameters of liver damage (alanine aminotransferase (ALT) and aspartate aminotransferase (AST)) and liver function (albumin and total protein) in fibrotic mice treated with dendrimer–graphene nanostars linked to mannitol (DGNS-Man) or GW1929 (DGNS-GW); Figure S1: Immunofluorescent staining of pro-inflammatory M1-like markers (NOS2 and COX-2) and anti-inflam-matory M2-like markers (MRC1 and ARG1) in the liver of in fibrotic mice treated with dendrimer-graphene nanos-tars linked to mannitol (DGNS-Man) or GW1929 (DGNS-GW). Scale bar: 250 μm.
