# Supplementary material for: PPAR-γ Agonist GW1929 Targeted to Macrophages with Dendrimer–Graphene Nanostars Reduces Liver Fibrosis and Inflammation
Source: Pharmaceutics. 2023 May 10;15(5):1452. doi: 10.3390/pharmaceutics15051452 (PMC10223394; doi:10.3390/pharmaceutics15051452)
Supplement: Supplementary file 1 [file pharmaceutics-15-01452-s001.zip › pharmaceutics-2343656-supplementary.pdf]

pharmaceutics

MDPI

Article

PPAR-γ Agonist GW1929 Targeted to Macrophages with Dendrimer–Graphene Nanostars Reduces Liver Fibrosis and Inflammation

Alazne Moreno-Lanceta [1,2], Mireia Medrano-Bosch [1], Blanca Simón-Codina [1], Montserrat Barber-Gonzá lez[1], Wladimiro Jiménez [1,2] and Pedro Melgar-Lesmes [1,2,3,*]

1 Department of Biomedicine, School of Medicine, University of Barcelona, 08036 Barcelona, Spain; amorenol@recerca.clinic.cat (A.M.-L.)
2 Biochemistry and Molecular Genetics Service, Hospital Clínic Universitari, Instituto de Investigaciones Biomédicas August Pi i Sunyer (IDIBAPS), Centro de Investigación Biomédica en Red de Enfermedades Hepáticas y Digestivas (CIBERehd), 08036 Barcelona, Spain
3 Institute for Medical Engineering and Science, Massachusetts Institute of Technology, Cambridge, MA 02139, USA
* Correspondence: pmelgar@ub.edu; Tel.: +34-934020294

Table S1. Serum parameters of liver damage (alanine aminotransferase (ALT) and aspartate aminotransferase (AST)) and liver function (albumin and total protein) in fibrotic mice treated with dendrimer-graphene nanostars linked to mannitol (DGNS-Man) or GW1929 (DGNS-GW).

Serum parameter	DGNS-Man (N=6)	DGNS-GW (N=6)
ALT (U/L)	37.13 ± 9.12	40.46 ± 5.46
AST (U/L)	277.8 ± 155	332.4 ± 66.87
Albumin (g/L)	27.05 ± 0.22	27.19 ± 0.37
Total protein (g/L)	49.53 ± 0.77	47.65 ± 0.89

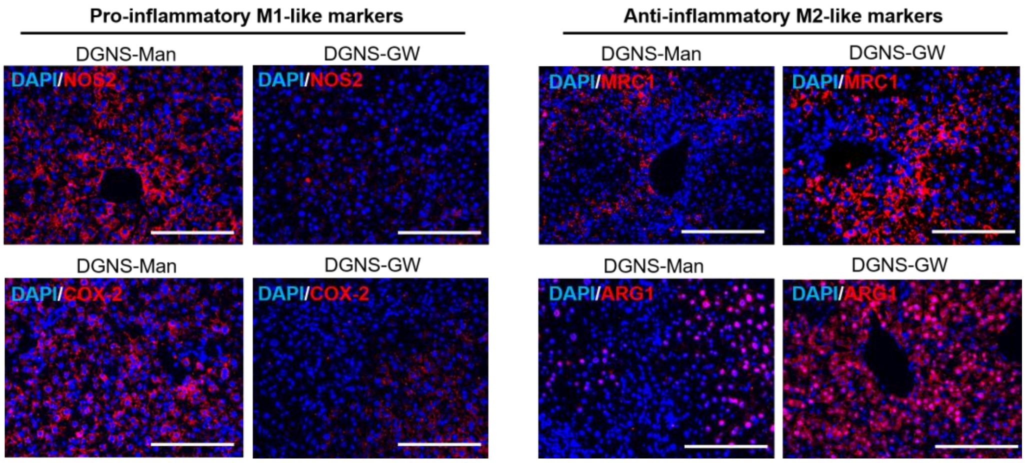

Figure S1. Immunofluorescent staining of pro-inflammatory M1-like markers (NOS2 and COX-2) and anti-inflammatory M2-like markers (MRC1 and ARG1) in the liver of in fibrotic mice treated with dendrimer-graphene nanostars linked to mannitol (DGNS-Man) or GW1929 (DGNS-GW). Scale bar: 250 µm.
